# Activation of c-jun N-terminal kinase in spinal cord contributes to breast cancer induced bone pain in rats

**DOI:** 10.1186/1756-6606-5-21

**Published:** 2012-06-09

**Authors:** Xiao-Wei Wang, Shan Hu, Qi-Liang Mao-Ying, Qian Li, Chang-Jiang Yang, Hui Zhang, Wen-Li Mi, Gen-Cheng Wu, Yan-Qing Wang

**Affiliations:** 1 Department of Integrative Medicine and Neurobiology, State Key Laboratory of Medical Neurobiology, Shanghai Medical College; Institute of Acupuncture Research (WHO Collaborating Center for Traditional Medicine), Fudan University, P.O. Box 291, 138 Yi Xue Yuan Road, Shanghai, 200032, China

**Keywords:** c-Jun N-terminal kinase, Cancer-induced bone pain, Spinal cord, Rats

## Abstract

**Background:**

The most frequent pain in patients with metastatic breast and prostate cancer is bone pain, which can be severe and difficult to treat. The mechanisms underlying this pain remain unclear. Here we investigated the role of c-jun N-terminal kinase (JNK) pathway in the spinal cord in cancer-induced bone pain (CIBP).

**Results:**

In this study, we used an established rat CIBP model to investigate the possible role of JNK activation in the spinal cord. After intra-tibial inoculation with Walker 256 rat mammary gland carcinoma cells, the rats displayed mechanical allodynia on day 5, which lasted to day 16. The activation of JNK in neurons and astrocytes in the spinal cord was found on day 12 and day 16 after intra-tibial inoculation with carcinoma cells. A single intrathecal injection with JNK inhibitor SP600125 by lumbar puncture attenuated mechanical allodynia on day 12, and repeated intrathecal injection of SP600126 from day 10 to day 14 had a cumulative analgesic effect on CIBP.

**Conclusions:**

Taken together, our results demonstrated for the first time that JNK activation in the spinal cord is required in the maintenance of CIBP. Inhibition of the spinal JNK pathway may provide a new therapy for CIBP management.

## Background

The c-jun N-terminal kinase (JNK) is an evolutionarily conserved sub-group of mitogen-activated protein kinases (MAPK) that participates in survival signaling, apoptosis and pain [1-3]. The JNK family is encoded by three genes: jnk1, jnk2 and jnk3. Recent studies have demonstrated that JNK1 and JNK2 activation play important roles in the development and maintenance of chronic pain [4]; JNK3 has different functions from JNK1 and JNK2 and has been reported to participate in apoptosis in the brain. JNK activation is mediated by the dual phosphorylation on Thr and Tyr by two MAPK kinases (MKK4/7), and several transcriptional factors can be regulated by JNK activation [5]. JNK1/2 was shown to be activated in the spinal cord at 6 h after intra-plantar injection of complete Freund’s adjuvant (CFA) [6] and at day 3 after spinal nerve ligation (SNL) [7]. Moreover, intrathecal injection of JNK inhibitor SP600125 decreased pain behavior in animals with inflammatory pain, neuropathic pain and skin cancer pain [8-10].

Cancer-induced bone pain (CIBP) is a severe problem for patients with end-stage cancer. The preferential metastasis of cancer cells to bone disrupts the process of bone remodeling and results in lesions that cause significant pain [11]. The model of bone cancer induced by intramedullary inoculation with tumor cells has been the most frequently encountered type of cancer-induced pain in cancer patients with bone metastasis [12]. Several animal models of CIBP have been developed recently, and these models contributed to our understanding of CIBP [13-15]. A widely used model of CIBP is induced by intra-tibial inoculation with Walker 256 rat mammary gland carcinoma cells [16-18]. Rats inoculated with carcinoma cells developed mechanical allodynia from day 5 as indicated by decreased paw withdrawal thresholds for the ipsilateral hind paw. Although basic research on the mechanisms of bone cancer pain has been developed in recent years, the mechanisms of CIBP remain unclear. Previous studies have indicated the important roles of MAPK, including the roles of extracellular signal-regulated kinases (ERK) and p38 in chronic pain [19,20]; however, the specific roles of JNK activation of bone cancer pain in the spinal cord remain unclear.

In this study, we found that JNK was activated at different time points in the spinal cord after intra-tibial inoculation with carcinoma cells; increased pJNK levels were co-expressed with NeuN (a neuron marker) and GFAP (Glial fibrillary acidic protein, a specific astrocyte marker) but not CD11b (microglia marker); a single intrathecal injection of JNK inhibitor SP600125 by lumbar puncture attenuated CIBP on day 12. These results suggested that JNK activation in the spinal cord participated in the development of CIBP.

## Results

### Sustained activation of pJNK1/2 in the spinal cord after intra-tibial inoculation with carcinoma cells

**Figure 1:**
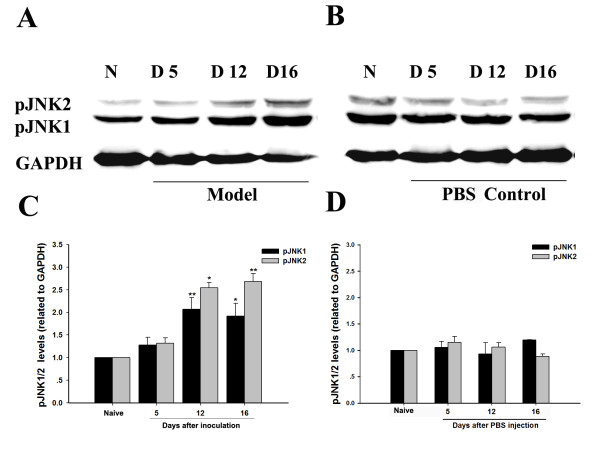
**Time course of pJNK upregulation on the ipsilateral side of L4-L5 spinal cord after intra-tibial inoculation with carcinoma cells**. **(A-B)** Representative Western blots of pJNK1/2 and GAPDH from one membrane. **(C-D)** Density of pJNK1/2 levels on the ipsilateral side of L4-L5 spinal cord. pJNK1/2 levels were normalized against GAPDH levels and expressed as fold increase, compared with naïve. (* p < 0.05 and ** p < 0.01, one-way ANOVA, Mean ± SEM n = 4)

pJNK1 and pJNK2 protein levels were detected on the ipsilateral side of L4-L5 spinal cord. We examined the expression of pJNK1/2 in either CIBP (Figure 1A) or a PBS control group (Figure 1B) at different time points after surgery. pJNK1/2 (46 kD, 52 kD) and GAPDH (36 kD) were detected in the same membrane. The levels of pJNK1/2 were not changed compared to the naïve group on day 5, day 12 or day 16 (Figure 1B, D) after the injection of PBS as a sham control. Compared to naïve rats, the pJNK1/2 protein levels were increased on the ipsilateral side of the spinal cord on day 12 and day 16 after intra-tibial inoculation with carcinoma cells (Figure 1C).

**Figure 2:**
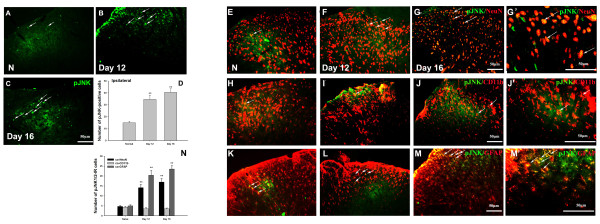
**Intra-tibial inoculation of carcinoma cells induced persistent JNK activation on the ipsilateral side of L4-L5 spinal cord**. **(A-C)** Time course of pJNK activation in ipsilateral side of L4-L5 spinal cord. **(D)** Quantitative measurement of pJNK-IR cells in the superficial dorsal horn (laminas I- III). **(E-M)** Double immunofluorescence of pJNK (green) with NeuN (red, E-G), CD11b **(H-J)** and GFAP (red, K-M) respectively. (N) Statistic analysis of pJNK1/2-IR cells co-expressed with NeuN, CD11b and GFAP. Scale bars: 50 μm. (G’, J’, M’) High magnification image of **G, J** and **M**. Scale bars: 50 μm. (** p < 0.01 vs Naive, one-way ANOVA, mean ± SEM, *n* = 4)

The number of pJNK positive cells was also increased by single-stained immunofluorescence on day 12 and day 16 after inoculation with carcinoma cells (Figure 2A-D). We then determined the cellular localization of pJNK1/2 in naïve and model animals (Figure 2E-M). Double immunofluorescence results showed that a small number of pJNK1/2-IR cells were double labeled with NeuN, CD11b and GFAP, indicating that pJNK1/2 was expressed in neurons, microglia and astrocytes in naïve rats (Figure 2E, H, K). A significant increase in the number of pJNK1/2-IR neurons and astrocytes was found on day 12 and day 16 in ipsilateral spinal cord after intra-tibial inoculation with carcinoma cells as compared to the naïve condition, but the number of pJNK1/2-IR microglia was not changed at any time point after intra-tibial inoculation with carcinoma cells (Figure 2N).

### Analgesic effects of intrathecal JNK inhibitor SP600125

**Figure 3:**
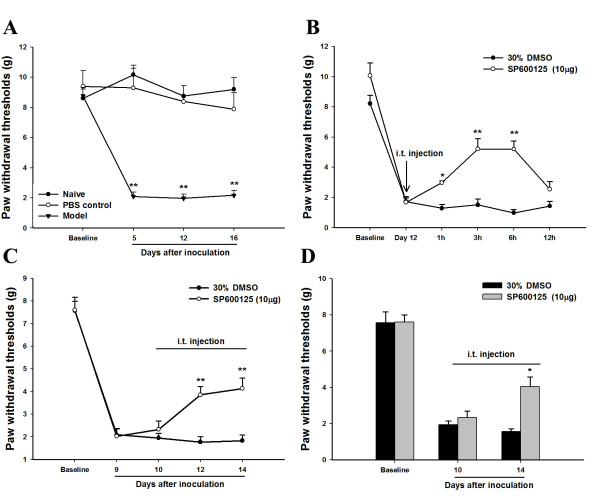
**The analgesic effect of JNK inhibitor SP600125 on the nociceptive response to mechanical stimulations.(A)** The paw withdrawal thresholds of ipsilateral side were significiantly decreased from day 5 until day 16. **(B)** The effect was tested immediately after a single intrathecal injection of SP600125 on day 12 after intra-tibial inoculation with carcinoma cells. **(C)** The accumulative (therapeutic) effect was tested 12 h after intrathecal injection of SP600125 on days 10, 12 and 14 after the intra-tibial inoculation of carcinoma cells. **(D)** The accumulative (therapeutic) effect was tested 24 h after intrathecal injection of SP600125 on day 10 and 14 after the intra-tibial inoculation of carcinoma cells. (* p < 0.05 and ** p < 0.01, compared to control, one-way ANOVA, Mean ± SEM *n* = 8)

The CIBP rats displayed significant decreases in mechanical thresholds on day 5, day 12 and day 16 after intra-tibial inoculation with carcinoma cells as compared to naïve rats or sham control rats injected with intra-tibial PBS (Figure 3A). We sought to assess whether the activation of JNK contributed to the mechanical allodynia induced by intra-tibial inoculation with carcinoma cells. A single intrathecal injection of SP600125, which respectively inhibited JNK phosphorylation, induced an increase in paw withdrawal thresholds at 1 h; this effect lasted for 6 h (Figure 3B). Furthermore, the CIBP rats received a repeated daily intrathecal injection of SP600125 from day 10 to 14 after intra-tibial inoculation with carcinoma cells. After 3 intrathecal injections of SP600125, the analgesic effect of SP600125 was observed to last for 12 h, while there was no analgesic effect of SP600125 on 12 h after a single injection (Figure 3C). After 5 daily intrathecal injections of SP600125, the analgesic effect of SP600125 was observed to last for 24 h (Figure 3D). Intrathecal injection of 30% DMSO had no effect on mechanical allodynia at any time point throughout the experiment.

## Discussion

In this study, we demonstrated JNK activation in neurons and astrocytes of the spinal cord after intra-tibial inoculation with carcinoma cells. A single intrathecal injection of JNK inhibitor SP600125 could attenuate bone cancer-induced mechanical allodynia. Interestingly, the repeated injection of SP600125 showed an accumulative analgesic effect. For example, the analgesic effect of SP600125 lasted up to 12 h after the previous injection when administered as repeated injections over 3 days and for 24 h when administered as repeated injections over 5 days.

Primary tumors including breast and prostate tumors have a particular propensity for metastasis to bone. Metastatic bone disease, especially bone pain, has a significant impact on the quality of life in patients with cancer. Despite the currently available therapies, CIBP is difficult to relieve and often associated with significant side effects [21-24]. Advances in treatment of CIBP require new insights into the mechanisms that initiate and maintain this type of serious pain. The animal model we used in this study was an established model of CIBP that was suitable for studying the clinical problem of CIBP. Analysis of bone destruction by radiographic scoring and the behavioral measurement of pain using the von Frey hair test indicated that intra-tibial inoculation with Walker 256 mammary gland carcinoma cells in the induced bone pain model caused severe and progressive pain [16,17]. In this study, the mechanical allodynia was observed on day 5, day 12 and day 16 after intra-tibial inoculation with carcinoma cells, but injection with PBS had no effect on paw-withdrawal thresholds. Clohisy found that no pain was observed when the malignancy was grown in soft tissue [22]. Thus, our results indicate that at the level of peripheral tissue, the tumor-induced bone destruction and the presence of tumor cells contributed to pain.

Among the multiple mechanisms of chronic pain, the role of MAPK activation included ERK, p38, and JNK in central sensitization has been investigated in recent years [20,25]. For example, JNK has been found to be activated in spinal astrocytes but not in neurons or microglia after spinal nerve ligation (SNL) and inflammation [6,26]. In our study, after intra-tibial inoculation with carcinoma cells, increased levels of pJNK were found not only in astrocytes but also in neurons in the spinal cord on day 12 and day 16. Although the mechanical thresholds were decreased on day 5 after intra-tibial inoculation with carcinoma cells, the pJNK levels were not changed compared to the naïve group at the early stage (< 5 D). Interestingly, the results were clearly different from those observed for inflammatory pain or neuropathic pain. Several studies have found that JNK1 in spinal astrocytes was required in inflammatory pain and neuropathic pain condition. Besides, CFA-induced inflammatory pain was attenuated in mice lacking JNK1 but not JNK2 [6,7]. In our results both pJNK1 and pJNK2 were increased in spinal cord, and inhibition of JNK by SP600125 attenuated the mechanical allodynia in bone cancer induced pain model. The selective JNK1 inhibitor and JNK2 inhibitor are needed to find the possible difference in the roles of JNK1 and JNK2 in further study. The differences between CIBP, inflammatory pain and neuropathic pain have been mentioned in a previous study that indicated that CIBP results in a unique pain state [27].

Several reasons account for the increased pJNK level, including the variation in levels of proinflammatory cytokines such as TNF-α, IL-1β and IL-6. It has been well accepted that after nerve injury, levels of proinflammatory cytokines increased in the spinal cord and became the primary activators of the JNK pathway [4,28,29]. Several studies have found the up-regulation of TNF-α, IL-1β and IL-6 in the spinal cord in the CIBP model [30,31]. Thus, after intra-tibial inoculation with carcinoma cells, it is probable that the increased release of proinflammatory cytokines induced JNK activation in the spinal cord. It is well known that NMDA receptors participate in the development of morphine tolerance and chronic pain [32]. Guo et al. has found that a noncompetitive NMDA receptor antagonist MK-801 not only decreased the expression of NR2B but also reduced the level of JNK activation in the spinal cord. This suggested that the spinal JNK activation in the context of morphine dependence in rats was N-methyl-D-aspartate (NMDA) receptor dependent [33]. The activation of NMDA receptors in the spinal cord of CIBP model animals has been reported in many studies [34-36]; thus, we suppose that the JNK activation in the spinal cord after intra-tibial inoculation with carcinoma cells may be induced by increased expression of NMDA receptors.

Previous studies have demonstrated that intrathecal injection of the JNK inhibitor SP600125 induced significant decreases in nociceptive behavior in inflammatory pain and neuropathic pain [6,7]. In our study, we also found that the JNK inhibitor SP600125 reversed CIBP. It remains to be investigated how JNK inhibition in the spinal cord regulates pain. It was reported that transcription factors such as c-jun, Elk-1, p53 and ATF-2 were shown to be regulated by JNK activation, which subsequently induced gene expression that contributed to pain sensitization [7,37].

## Conclusions

In summary, our results demonstrated that intra-tibial inoculation with carcinoma cells induced obvious pain behavior in rats and caused JNK phosphorylation in the neurons and astrocytes of the spinal cord. Furthermore, the inhibition of JNK by SP600125 attenuated mechanical allodynia, providing a new method to control CIBP.

## Methods

### Animals

Adult female Wistar rats weighing 160–200 g were used in all experiments. All animals were kept under controlled conditions (a temperature-controlled room (22 ± 0.5°C), a 12:12 h light cycle (07:00–19:00 h light), and with unrestricted free access to food and water). All animal experiments followed the guidelines of the International Association for the Study of Pain (IASP) [38]. Efforts were made to reduce the number of animals used in the experiment.

### Surgical procedures

Walker 256 rat mammary gland carcinoma cells were used in the experiment. Suspensions of 1 × 10^8^/ml tumor cells in PBS were prepared as previously described [13,16,17,39]. After the animals were anesthetized with sodium pentobarbital (i.p. 50 mg/kg), 4 × 10^5^ cells in 4 μl 0.01MPBS were injected into the right tibias of female Wistar rats. Briefly, the Walker 256 carcinoma cells were obtained from an ascetic tumor-bearing rat, washed with PBS 3 times, and then diluted to 1 × 10^8^/ml during the last wash. Bilateral superficial incisions were made in the skin overlying the patella after disinfection with 70% v/v ethanol in order to expose the tibia head with minimal damage. After Walker 256 carcinoma cells were prepared, 4 μl cells followed by 4 μl of absorbable gelatin sponge dissolved in saline were slowly injected into the right tibia cavity of each rat using a 10-μl microinjection syringe. The syringe was left in place for an additional 2 min to prevent the carcinoma cells from leaking out along the injection track. The injection site was closed using bone wax while the syringe was removed to prevent tumor cells overflow. The sham group rats were treated in the same way and injected with 4 μl PBS instead of tumor cells.

### Intrathecal drugs

The JNK inhibitor SP600125 (1,9-Pyrazoloanthrone) was purchased from Calbiochem (San Diego, California, USA). SP600125 stock solution was prepared in DMSO at a concentration 20 μg/μl and stored at -20°C until use. The concentration used for the study was 1 μg/μl, which was freshly prepared with a final DMSO concentration of 30%. Ten μg were used in the experiment, and the control group was treated with the same amount of DMSO. The dose of drug used in the experiment was chosen based on the previous research [7,8]. Rats were anesthetized with 2% isoflurane. After the lumbar region was shaved and sterilized with 75% ethanol, animals were given a lumbar puncture at the L5-6 interspace using a 0.5-inch, 30-gauge needle. Then the drug was delivered to the CSF through the needle [40]. SP600125 was given once on day 12; for testing the addictive effect of SP600125, the drug was given daily from day 10 to day 14 after carcinoma cell inoculation.

### Western blot

The spinal cord segments were removed and immediately placed in liquid nitrogen to freeze quickly. The ipsilateral L4-L5 segments were quickly removed and homogenized in an SDS sample buffer (25 mM Tris–HCl, pH 7.6, 150 mM NaCl, 0.1% SDS, 1 mM each, PMSF, NaF, NaVO_3_, 1 μg/ml each, leupeptin, pepstatin, aprotinin), followed by centrifugation at 12000 g for 20 min. The protein concentration of the supernatant was determined by BCA Protein Assay Kit (Pierce, Rockford, USA). Thirty μg protein was boiled for 3 min at 100°C with an appropriate volume of 5× SDS-PAGE sample loading buffer (250 mM Tris–HCl, pH 6.8, 5% 2-mercaptoethanol, 10% SDS, 0.5% Bromophenol Blue, 50% glycerol). Samples were loaded into each lane of a 10% SDS-PAGE gel. The membrane was blocked by 5% bovine serum albumin in TBS-T (50 mM Tris–HCl, pH 7.6, 140 mM NaCl, 0.1% Tween 20) at 4°C overnight. Primary and secondary antibodies were also diluted in blocking solution at room temperature for 3 h. Blots were developed in ECL (Pierce, Rockford, US) solution for 3 min and exposed onto Kodak X-OMAT AR Film (Eastman Kodak, Rochester, US) for 3 min. The antibodies used were rabbit anti-phosphorylation SAPK/JNK (Thr183/Tyr185) antibody (1:1000, Cell signaling Technology, Massachusetts, US), HRP-anti-rabbit antibody(1:1000, Santa Cruz, California, US)and mouse HRP-anti-GAPDH (1:5000, Santa Cruz, California, US), which was used as a loading control in all Western blots. Densitometry analysis of pJNK1/2 bands and GAPDH bands were performed using Syngene software (GeneGnome, Syngene, Maryland, US). The same size square was drawn around each band to measure the density and subtract the background near that band. pJNK1/2 levels were normalized against GAPDH levels and expressed as fold increase, compared to the naive condition.

### Immunofluorescence

Four rats from each group were used in the experiment. The L4–L5 spinal segments were removed, post-fixed, frozen and cut on a freezing microtome (Leica 2000, Germany) at 30-μm thickness. The sections were washed three times and blocked with 4% donkey serum in 0.3% Triton X-100 for 1 h at 37°C and then incubated with primary antibodies at 4°C overnight and with secondary antibodies at room temperature for 1 h. The primary antibodies used were rabbit anti-phosphorylation SAPK/JNK (Thr183/Tyr185) (1:400, Cell signaling Technology, Massachusetts, US), mouse anti-NeuN (neuronal marker, 1:1000, Chemicon, California, US), mouse anti-GFAP (astrocyte marker, 1:1000, Sigma-Aldrich, Missouri, US) and mouse anti-CD11b (microglia marker, 1:400, Chemicon, California, US). The secondary antibodies used were Cy3-conjugated affinity purified goat anti-mouse (1:1000, Millipore, CA) and Alexa Fluor 488-labeled donkey anti-rabbit (1:200, Invitrogen, Eugene, Oregon, USA). The stained sections were examined with a Leica fluorescence microscope. The number of pJNK-IR cells was counted in lamina I-II and lamina III-IV of the ipsilateral spinal dorsal horn that captured by using a computerized image analysis system (Leica Qwin 500). The specificity for pJNK antibody we used was confirmed by the lack of staining in the absence of primary antibody, and also specific bands on the membrane in Western blots. Based on the intensity of the staining, a threshold was chosen from the spinal cord of naïve animal to judge the signal was true or false. A signal below the threshold was considered as false positive. The backgrounds of the cell free area nearby the positive pJNK-IR and the depth lamina were subtracted. The number of pJNK-IR cells was recorded after removing the repeated count. For counting the double staining, the pJNK-IR neurons were determined by the distinct morpho-logy from glia cells and the colocalization with NeuN. The pJNK-IR glia cells were determined by the morphology and the colocalization with CD11b or GFAP. At least 4 rats from each group and each time point were analyzed. A minimum of 6 sections randomly selected from each rat were used in the experiment.

### Behavioral tests

Eight rats in each group were used in the experiment. The day of carcinoma cell inoculation was referred to as day 0. Mechanical allodynia was assessed using a von Frey hair filament (Stoelting, Wood Dale, Illinois, US) as previously described [16]. An ascending series of von Frey filaments with logarithmically incremental stiffness (0.40, 0.60, 1.4, 2.0, 4.0, 6.0, 8.0 and 15.0 g) were used in the experiment. The test began with the application of the 2.0 g von Frey filament. Each plantar surface of the hind paws was stimulated individually in the experiment. Each von Frey hair was held about 1–2 s, the positive response was defined as a withdrawal of hind paw or licking. We used a lower hair when the positive response was appeared, otherwise used the higher hair. After five more stimuli counted from the first change, a score was record. The final score was gotten by using the method described by Dixon which converted to a 50% von Frey threshold. Animals were habituated to the environment daily for at least 2 days before baseline testing. To test the paw withdrawal thresholds, animals were put into the experimental environment for 30 min before stimulation. The pre-drug baseline was assessed 1 h before intrathecal injection. All of the tests were performed with researchers blinded with respect to the drugs injected.

### Statistical analysis

All data were presented as mean ± standard error of the mean (SEM). The statistical significance of differences between groups was analyzed with one-way ANOVA following the Bonferroni post-test or with Student’s *t*-test. *p* < 0.05 was set as the threshold of significance.

## List of abbreviations

CIBP: cancer induced bone pain; JNK: c-jun N-terminal kinase; MAPK: mitogen-activated protein kinases; ERK: extracellular signal-regulated kinases; IR: immunoreactivity; IL: interleukin.

## Competing interests

The authors declare that they have no competing interests.

## Authors’ contributions

XWW carried out all the experiments, performed the statistical analysis and drafted the manuscript. HZ, QL, SH, CJY contributed to the establishment of animal models and animal care directed by QLMY. YQW contributed to the design of the study and the editing of the manuscript. GCW, WLM and QLMY participated in the design of the study and the review of the manuscript. All authors have read and approved the final manuscript.

## References

[B1] Lin A Dibling B The true face of JNK activation in apoptosis Aging Cell 2002 1 2 112 116 10.1046/j.1474-9728.2002.00014.x 12882340

[B2] Cavalli V Kujala P Klumperman J Goldstein LS Sunday Driver links axonal transport to damage signaling J Cell Biol 2005 168 775 787 10.1083/jcb.200410136 15738268 PMC2171809

[B3] Wang W Mei XP Wei YY Zhang MM Zhang T Wang W Xu LX Wu SX Li YQ Neuronal NR2B-containing NMDA receptor mediates spinal astrocytic c-Jun N-terminal kinase activation in a rat model of neuropathic pain Brain Behav Immun 2011 25 1355 1366 10.1016/j.bbi.2011.04.002 21496481

[B4] Gao YJ Ji RR Activation of JNK pathway in persistent pain Neurosci Lett 2008 437 180 183 10.1016/j.neulet.2008.03.017 18455869 PMC2486445

[B5] Whitmarsh AJ Davis RJ Transcription factor AP-1 regulation by mitogen-activated protein kinase signal transduction pathways J Mol Med (Berl) 1996 74 589 607 10.1007/s001090050063 8912180

[B6] Gao YJ Xu ZZ Liu YC Wen YR Decosterd I Ji RR The c-Jun N-terminal kinase 1 (JNK1) in spinal astrocytes is required for the maintenance of bilateral mechanical allodynia under a persistent inflammatory pain condition Pain 2010 148 309 319 10.1016/j.pain.2009.11.017 20022176 PMC2814908

[B7] Zhuang ZY Wen YR Zhang DR Borsello T Bonny C Strichartz GR Decosterd I Ji RR A peptide c-Jun N-terminal kinase (JNK) inhibitor blocks mechanical allodynia after spinal nerve ligation: respective roles of JNK activation in primary sensory neurons and spinal astrocytes for neuropathic pain development and maintenance J Neurosci 2006 26 3551 3560 10.1523/JNEUROSCI.5290-05.2006 16571763 PMC6673862

[B8] Hao J Liu MG Yu YQ Cao FL Li Z Lu ZM Chen J Roles of peripheral mitogen-activated protein kinases in melittin-induced nociception and hyperalgesia Neuroscience 2008 152 1067 1075 10.1016/j.neuroscience.2007.12.038 18329815

[B9] Gao YJ Cheng JK Zeng Q Xu ZZ Decosterd I Xu X Ji RR Selective inhibition of JNK with a peptide inhibitor attenuates pain hypersensitivity and tumor growth in a mouse skin cancer pain model Exp Neurol 2009 219 1 146 155 10.1016/j.expneurol.2009.05.006 19445931 PMC2728781

[B10] Gao YJ Zhang L Samad OA Suter MR Yasuhiko K Xu ZZ Park JY Lind AL Ma Q Ji RR JNK-induced MCP-1 production in the spinal cord astrocytes contributes to central sensitization and neuropathic pain J Neurosci 2009 29 4096 4108 10.1523/JNEUROSCI.3623-08.2009 19339605 PMC2682921

[B11] Brown JE Sim S Evolving role of bone biomarkers in castration-resistant prostate cancer Neoplasia 2010 12 685 696 20824045 10.1593/neo.10610 PMC2933689

[B12] Mercadante S Malignant bone pain: pathophysiology and treatment Pain 1997 69 1 18 10.1016/S0304-3959(96)03267-8 9060007

[B13] Medhurst SJ Walker K Bowes M Kidd BL Glatt M Muller M Hattenberger M Vaxelaire J O’Reilly T Wotherspoon G Winter J Green J Urban L A rat model of bone cancer pain Pain 2002 96 129 140 10.1016/S0304-3959(01)00437-7 11932069

[B14] Menendez L Lastra A Fresno MF Llames S Meana A Hidalgo A Baamonde A Initial thermal heat hypoalgesia and delayed hyperalgesia in a murine model of bone cancer pain Brain Res 2003 969 102 109 10.1016/S0006-8993(03)02284-4 12676370

[B15] De Ciantis PD Yashpal K Henry J Singh G Characterization of a rat model of metastatic prostate cancer bone pain J Pain Res 2010 3 213 221 21197325 10.2147/JPR.S14209 PMC3004636

[B16] Mao-Ying QL Zhao J Dong ZQ Wang J Yu J Yan MF Zhang YQ Wu GC Wang YQ A rat model of bone cancer pain induced by intra-tibia inoculation of Walker 256 mammary gland carcinoma cells Biochem Biophys Res Commun 2006 345 1292 1298 10.1016/j.bbrc.2006.04.186 16725112

[B17] Zhao J Pan HL Li TT Zhang YQ Wei JY Zhao ZQ The sensitization of peripheral C-fibers to lysophosphatidic acid in bone cancer pain Life Sci 2010 87 120 125 10.1016/j.lfs.2010.05.015 20553953

[B18] Tong W Wang W Huang J Ren N Wu SX Li YQ Spinal high-mobility group box 1 contributes to mechanical allodynia in a rat model of bone cancer pain Biochem Biophys Res Commun 2010 395 572 576 10.1016/j.bbrc.2010.04.086 20399746

[B19] Svensson CI Medicherla S Malkmus S Jiang Y Ma JY Kerr I Brainin-Mattos J Powell HC Luo ZD Chakravarty S Dugar S Higgins LS Protter AA Yaksh TL Role of p38 mitogen activated protein kinase in a model of osteosarcoma-induced pain Pharmacol Biochem Behav 2008 90 664 675 10.1016/j.pbb.2008.05.016 18584857

[B20] Lee Y Pai M Brederson JD Wilcox D Hsieh G Jarvis MF Bitner RS Monosodium iodoacetate-induced joint pain is associated with increased phosphorylation of mitogen activated protein kinases in the rat spinal cord Mol Pain 2011 7 39 10.1186/1744-8069-7-39 21599960 PMC3120677

[B21] Foley KM Advances in cancer pain Arch Neurol 1999 56 413 417 10.1001/archneur.56.4.413 10199328

[B22] Clohisy DR Mantyh PW Bone cancer pain Clin Orthop Relat Res 2003 415 Suppl S279 S288 14600620 10.1097/01.blo.0000093059.96273.56

[B23] Mantyh PW Cancer pain and its impact on diagnosis, survival and quality of life Nat Rev Neurosci 2006 7 797 809 16988655 10.1038/nrn1914

[B24] Woodward EJ Coleman RE Prevention and treatment of bone metastases Curr Pharm Des 2010 16 2998 3006 10.2174/138161210793563581 20722624

[B25] Ji RR 4th Gereau RW Malcangio M Strichartz GR MAP kinase and pain Brain Res Rev 2009 60 135 148 10.1016/j.brainresrev.2008.12.011 19150373 PMC2666786

[B26] Mei XP Zhang H Wang W Wei YY Zhai MZ Wang W Xu LX Li YQ Inhibition of spinal astrocytic c-Jun N-terminal kinase (JNK) activation correlates with the analgesic effects of ketamine in neuropathic pain J Neuroinflammation 2011 8 6 10.1186/1742-2094-8-6 21255465 PMC3033337

[B27] Honore P Rogers SD Schwei MJ Salak-Johnson JL Luger NM Sabino MC Clohisy DR Mantyh PW Murine models of inflammatory, neuropathic and cancer pain each generates a unique set of neurochemical changes in the spinal cord and sensory neurons Neuroscience 2000 98 585 598 10.1016/S0306-4522(00)00110-X 10869852

[B28] Manjavachi MN Motta EM Marotta DM Leite DF Calixto JB Mechanisms involved in IL-6-induced muscular mechanical hyperalgesia in mice Pain 2010 151 345 355 10.1016/j.pain.2010.07.018 20709454

[B29] Gao YJ Zhang L Ji RR Spinal injection of TNF-α-activated astrocytes produces persistent pain symptom mechanical allodynia by releasing monocyte chemoattractant protein-1 Glia 2010 58 1871 1880 10.1002/glia.21056 20737477 PMC2951489

[B30] Gu X Zheng Y Ren B Zhang R Mei F Zhang J Ma Z Intraperitoneal injection of thalidomide attenuates bone cancer pain and decreases spinal tumor necrosis factor-α expression in a mouse model Mol Pain 2010 6 64 10.1186/1744-8069-6-64 20923560 PMC2959022

[B31] Yao M Chang XY Chu YX Yang JP Wang LN Cao HQ Liu MJ Xu QN Antiallodynic effects of propentofylline Elicited by interrupting spinal glial function in a rat model of bone cancer pain J Neurosci Res 2011 89 1877 1886 10.1002/jnr.22711 21812015

[B32] Muir WW NMDA receptor antagonists and pain: ketamine Vet Clin North Am Equine Pract 2010 26 565 578 10.1016/j.cveq.2010.07.009 21056300

[B33] Guo RX Zhang M Liu W Zhao CM Cui Y Wang CH Feng JQ Chen PX NMDA receptors are involved in upstream of the spinal JNK activation in morphine antinociceptive tolerance Neurosci Lett 2009 467 95 99 10.1016/j.neulet.2009.10.013 19818835

[B34] Zhang RX Liu B Li A Wang L Ren K Qiao JT Berman BM Lao L Interleukin 1beta facilitates bone cancer pain in rats by enhancing NMDA receptor NR-1 subunit phosphorylation Neuroscience 2008 154 1533 1538 10.1016/j.neuroscience.2008.04.072 18554806 PMC2495055

[B35] Yanagisawa Y Furue H Kawamata T Uta D Yamamoto J Furuse S Katafuchi T Imoto K Iwamoto Y Yoshimura M Bone cancer induces a unique central sensitization through synaptic changes in a wide area of the spinal cord Mol Pain 2010 6 38 10.1186/1744-8069-6-38 20602757 PMC3020802

[B36] Gu X Zhang J Ma Z Wang J Zhou X Jin Y Xia X Gao Q Mei F The role of N-methyl-D-aspartate receptor subunit NR2B in the spinal cord in cancer pain Eur J Pain 2010 14 496 502 10.1016/j.ejpain.2009.09.001 19815434

[B37] Bogoyevitch MA Kobe B Uses for JNK: the many and varied substrates of the c-Jun N-terminal kinases Microbiol Mol Biol Rev 2006 70 1061 1095 10.1128/MMBR.00025-06 17158707 PMC1698509

[B38] Zimmermann M Ethical guidelines for investigations of experimental pain in conscious animals Pain 1983 16 109 110 10.1016/0304-3959(83)90201-4 6877845

[B39] Liu S Liu WT Liu YP Dong HL Henkemeyer M Xiong LZ Song XJ Blocking EphB1 receptor forward signaling in spinal cord relieves bone cancer pain and rescues analgesic effect of morphine treatment in rodents Cancer Res 2011 71 4392 4402 10.1158/0008-5472.CAN-10-3870 21555368

[B40] Xu JJ Walla BC Diaz MF Fuller GN Gutstein HB Intermittent lumbar puncture in rats: A novel method for the experimental study of opioid tolerance Anesth Analg 2006 103 714 720 10.1213/01.ane.0000226100.46866.ea 16931686

